# Phylogenetic Diversity and Extracellular Enzymatic Activities of Yeasts and Yeast-Like Fungi Isolated From *Qualea grandiflora* (Vochysiaceae) in Cerrado Areas in Northern Minas Gerais, Brazil

**DOI:** 10.1155/ijm/2663995

**Published:** 2025-07-15

**Authors:** Jaqueline Silva Vieira, Dailane Amaral de Almeida, Rodrigo Oliveira Pessoa, Magno Augusto Zazá Borges, Henrique Maia Valério

**Affiliations:** Department of General Biology, Montes Claros State University, Montes Claros, Minas Gerais, Brazil

**Keywords:** Brazilian savannah, new species, secretome, selective enzymes, wildflowers, yeast diversity

## Abstract

The Brazilian savannah, Cerrado, a Brazilian phytophysiognomy, is an entire biome that contains as well as other Brazilian ecosystems, a vast biodiversity of microorganisms associated with native plants. Plant species of the Cerrado have attracted attention due to the still limited knowledge regarding their associated microbiota and the possible applications of these microorganisms. Considering that wildflowers are rich reservoirs of yeast diversity, the present study isolated 58 yeast strains from flowers of *Qualea grandiflora* from two areas of Cerrado *sensu stricto* in northern Minas Gerais, Brazil. The isolates were evaluated for producing extracellular enzymes in cellulase, amylase, xylanase, protease, tannase, and pectinase tests. We used the YNB agar diffusion method (Difco) supplemented with specific substrates for each enzyme. The strains were identified by analyzing the sequences of the D1/D2 region of the large subunit (26S) rRNA gene and compared to the sequences deposited on GenBank. Fifty-eight strains were isolated, and 14 genera were identified, of which 18 species were yeasts, two species were yeast-like fungi, and three were yeast-like strains to which it was not yet possible to assign the species they belonged to. Among the identified species, the phylum Ascomycota predominated; exceptions were the isolates of the yeast *Papiliotrema laurentii* and the fungus *Anthracocystis heteropogonicola*, both belonging to the phylum Basidiomycota. In the enzymatic tests, 44.8% of the strains tested presented an enzymatic profile in solid medium, being capable of producing at least one of the enzymes studied, with the species *Coniochaeta rhopalochaeta* showing the greatest cellulolytic activity.

## 1. Background

The Cerrado is the second largest forest formation in Brazil, and it is one of the most biodiverse biomes on the planet [1]. It has been estimated that there are more than 11,620 vascular plant species endemic to the biome [2]. One such species is *Qualea grandiflora* Mart. (Vochysiaceae), one of the main woody plants of the Cerrado, has been found in more than 80% of studied areas [3, 4]. The species has economic and ecological importance for commercial, ornamental [5], reforestation [6], and medical applications [7]. In view of their morphophysiological characteristics, such as color and nectar supply, the flowers of *Q. grandiflora* can be classified as “mini-hotspots” or microbial islands, as they favor interrelationships with macro- and microorganisms and thus harbor a miniature model ecosystem ideal for the ecological study of microorganisms.

Fungi, although not belonging to fauna or flora, are closely associated with both and constitute a vast biological universe to be explored. These associations are even more frequent in tropical forests given the adaptability of fungi and the abundance of available microhabitats and nutrients. Hawksworth [8] estimated that for each species of vascular plant, there are on average six associated fungal species. From this perspective, the Cerrado would house 69,720 species of fungi. However, some studies have shown that these estimates are well below reality and that the true value is much higher [9, 10]. Despite efforts, the actual mycological diversity of Brazilian biomes remains little known, while the lack of incentives for basic systematic research on fungi has caused the description of fungal species to proceed slowly [11]. On the other hand, some research groups in Brazil and around the world have been focusing on the description of the taxonomic diversity of yeasts and their desirable characteristics from the point of view of biotechnological application based on studies of various plant species from the Brazilian Savanna and other different ecosystems, which have received special attention in recent times [12, 13].

Belonging to the Fungi kingdom, yeasts are a very diverse group and, due to their wide distribution, it is not uncommon for them to be found in association with plant parts, such as leaves, fruits, and flowers, and in animals, like insects [14, 15]. These insects play a fundamental role in the establishment of yeast communities associated with flowers since they are the main responsible for introducing and dispersing these microorganisms through the pollination and visitation processes for foraging [16, 17]. The plant parts, particularly fruits and flowers, although ephemeral, are considered excellent reservoirs for the establishment of yeast communities due to the amount of available simple sugars [18]. In addition, these microorganisms can act as a natural barrier for plants, protecting them from phytopathogens through the production of extracellular enzymes and secondary metabolites with antagonistic action [19–21]. These enzymes, meanwhile, can favor the ecological and physiological processes of the host plant, which can explain the cases in which yeasts establish themselves in various habitats.

Yeasts are microorganisms that arouse great interest in several areas, among which biotechnology stands out due to their productive efficiency compared to other fungal species. This is due to characteristics such as their accelerated growth and reproduction time without the need for an inducer, adaptation to wide ranges of acidic pH, relatively high concentrations of ethanol and sucrose (55%–60%), and because they are more efficient in making biochemical changes, that is, structural biochemical modification in different sugars (by invertases or isomerases) [22]. Thus, bioprospecting of these microorganisms is becoming more prominent within biotechnology not only to add value to materials for human consumption but also because it represents a more conscientious and efficient use of the planet's renewable resources. In addition, yeasts that are adapted to the heterogeneity of the Cerrado, such as variation in climate, soil, and rainfall, may have their own characteristics and peculiarities, which make them even more attractive to biotechnology and bioscience [11, 13, 23].

Considering that wild flowers are a little-explored microcosm in relation to mycological biodiversity, yeasts were isolated, and after isolation, they were identified by amplification and sequencing of the conserved phylogenetic markers of the D1/D2 domain of the large subunit (26S) of the rRNA gene, which allowed them to associate these yeasts with *Q. grandiflora* flowers. We also evaluated the potential of isolated strains to synthesize enzymes of industrial interest in order to elucidate one of the stages of the biochemical characterization of these microorganisms.

## 2. Materials and Methods

### 2.1. Characterization of Areas and Collection of Samples

**Figure 1:**
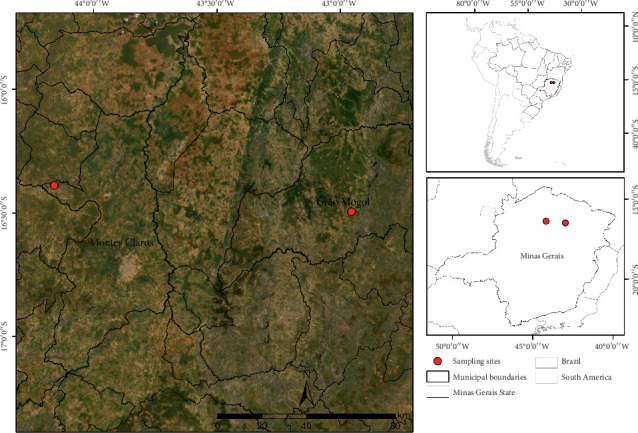
Map showing the sites where *Q. grandiflora* flowers were collected. Red dots correspond to the municipalities of Grão Mogol and Montes Claros, north of Minas Gerais, Brazil.

This study was conducted in two municipalities in northern Minas Gerais State, Brazil (Figure 1). The two collection areas originally corresponded to the ecosystems of Cerrado *sensu stricto*, with one area (Area I: 16°29⁣′40,93⁣^″^ S, 42°57⁣′19,16⁣^″^ W; altitude 825 m) located in Grão Mogol, a municipality whose climate is classified as humid subtropical (Cwa), while the other area (Area II: 16°23⁣′20,28⁣^″^ S, 44°9⁣′30,00⁣^″^ W; altitude 812 m) is located in Montes Claros, a municipality located in a region with a tropical savanna climate (Aw), according to the Köppen-Geiger classification [24].

In addition to climatic differences, there are noticeable differences in ecological and socioenvironmental aspects between the areas, including altitude, vegetation, and degree of anthropization. Area I is surrounded by a rural matrix with abandoned pasture areas, monocultures, and some fragments of preserved Cerrado. In contrast, Area II is located in a region close to an urban matrix, and the fragments of preserved vegetation are very small and have a higher degree of anthropization.

A total of 96 flowers of *Q. grandiflora* were collected in different stages of dehiscence, with 48 flowers in each area. The flowers were collected aseptically and deposited, immediately upon being collected, in Falcon tubes containing 25 mL of sterile saline–peptone solution (0.9% of NaCl and 1% peptone), supplemented with chloramphenicol (0.05%, *w*/*v*), for transport.

### 2.2. Isolation

Yeast isolates were obtained by shaking the Falcon tubes vigorously in a vortex shaker and then spreading 100 *μ*L of the liquid on plates containing YM agar medium (0.3% yeast extract, 0.3% malt extract, 0.5% peptone, 1.0% glucose, 2% agar, and 5 *μ*g/*μ*L of chloramphenicol), which were then incubated in a microbial growth oven at 28°C (±3°C) for 3–8 days. The growth of yeast colonies on the plates was observed during incubation, after which purification was performed by morphological typing using the streak-by-depletion technique on YEPD agar medium (1.0% yeast extract, 2.0% peptone, and 2.0% dextrose). The isolates were then kept refrigerated at 4°C until identification. The strains examined in this study are deposited in the Collection of Microorganisms and Cells of the Federal University of Minas Gerais (UFMG), Belo Horizonte, Minas Gerais, Brazil.

### 2.3. DNA Sequencing and Phylogenetic Analysis

For the extraction of DNA and amplification by polymerase chain reaction (PCR), standard methods were used, according to [25, 26], respectively. D1/D2 divergent domains were amplified by PCR using primers NL-1 (5 ‘GCATATCAATAAGCGGAGGAAAAG3') and NL-4 (5 ‘GGTCCGTGTTTCAAGAGGG3') and sequenced using BigDye Version 3.1 (Applied Biosystems, United States) in combination with the ABI 3730 automated sequencing system. Yeast isolates were grown in liquid YM culture medium for a period of 1–3 days. After growth, cells were precipitated by centrifugation of 1.5 mL of the culture. DNA was extracted by the chloroform/isoamyl alcohol method, using glass beads for mechanical lysis of the cells and extraction buffer (2% Triton X, 1% SDS, 100 mM NaCl, 10 mM Tris pH 8, 1 mM EDTA pH 8). The DNA fragment corresponding to the D1/D2 domain of the 26S ribosomal RNA gene was amplified by PCR using primers in a reaction with a final volume of 25 *μ*L containing 20–30 ng of template DNA, 20 pmol of each primer, 1.5 mM MgCl2, and 0.2 mM dNTPs (dATP, dCTP, dGTP, and dTTP). The PCR amplification criteria used were denaturation at 92°C for 40 s, annealing at 57°C for 1 min and 30 s, extension at 72°C for 2 min, and final extension at 72°C for 5 min. The amplified fragments were treated to remove impurities and unwanted materials. After treatment, the fragments were subjected to sequencing.

The resultant sequences were analyzed using the PHPH tool (http://asparagin.cenargen.embrapa.br/phph/) [27] for quality verification and then compared with sequences deposited in GenBank using the Basic Local Alignment Search Tool (BLAST) of the National Center for Biotechnology Information (NCBI) (http://www.ncbi.nlm.nih.gov) [28]. Alignment and phylogenetic relationships among sequences of the isolates and the sequences of GenBank were determined with the MEGA X program [29] using the maximum likelihood method with the Kimura-2-parameter evolutionary model and a discrete gamma distribution (+ G) with five categories, assuming that a certain fraction of sites is evolutionary invariable (+ I) [30]. Bootstrap analysis [31] to estimate confidence limits was based on 1000 replications. The nucleotide sequences obtained there were deposited in GenBank under the accession numbers given in Table 1.

### 2.4. Extracellular Enzyme Activities

The isolated yeasts were tested for their ability to degrade pectin, starch, carboxymethylcellulose (CMC), skim milk proteins (casein), xylan, and tannic acid using the agar diffusion method. For this procedure, the isolates were previously cultured at 25°C for 24–48 h on medium (to check for purity and metabolic activity). After growth, a portion of the colony was resuspended in a test tube containing 2 mL of sterile distilled water. Inocula standardization was performed using an optical density at 600 nm (OD600) of 0.05 of each yeast species suspension. After resuspending the cultures, 5 mL of each sample (in triplicate) was inoculated into plates containing YNB agar culture medium (Difco), enriched with specific carbon sources as mentioned above for the detection of each corresponding enzyme activity [32–35]. After inoculation, the plates were incubated from 5 to 8 days at 25°C, as some of the isolated yeasts (specimens) were not growing well at the initial temperature of 28°C, requiring adaptation to minimize sample loss. Verification of positive results was made by observing halos formed around the colonies, and negative results by their absence. The enzymatic activity for each microorganism was determined semiquantitatively using the enzymatic index (EI) represented by the equation: EI = dh/dc, where dh is the average diameter of the substrate hydrolysis halos and dc is the average diameter of the colonies, both measured and expressed in millimeters [34, 36].

## 3. Results and Discussion

### 3.1. Molecular Isolation and Identification

From the 96 samples from flowers of *Q. grandiflora*, a total of 58 yeast isolates were recovered: 56 yeasts and two yeast-like fungi. Flowers, in general, are seen as optimal niches for the proliferation and survival of microorganisms, especially yeasts, as they are rich in organic compounds such as monosaccharides, polysaccharides, and other carbon and nitrogen compounds, which favor microbiological growth [14]. In addition to physiology, flowers have a morphological apparatus that is evolutionarily attractive to insects and other pollinators and floral visitors. This characteristic, in addition to promoting the dispersion of yeasts, facilitates genetic exchange between established yeast communities.

The yeasts and yeast-like fungi recovered from the flowers of *Q. grandiflora* were identified by sequencing the D1/D2 region of the large subunit (26S) rRNA gene and comparing the sequences with those deposited in GenBank, where the values of identity and coverage are considered (Table 1).

The microbial community isolated from the flowers of *Q. grandiflora* was found to be composed of 18 different yeast species and two species of *yeast-like* fungi. Yeasts were represented by 14 genera, with *Candida* being the most prevalent with five species. The other genera represented by more than one species were *Meyerozyma* and *Wickerhamomyces*, both with two species. In addition to being represented by a greater number of species, the genus *Candida* also had a higher number of occurrences among the isolates, followed by the genera *Starmerella*, *Wickerhamomyces*, and *Coniochaeta*.

The *Candida* genus is represented by species that easily establish themselves in the most diverse functional niches, habitats, and hosts, as long as these locations offer the minimum conditions necessary for the survival and adaptation of this group of yeasts. Thus, the identification of more than one species of this genus among the identified Ascomycetic yeasts was not surprising, since the genus *Candida* incorporates a large group of yeasts from the same phylum, in many cases not even correlated based solely on taxonomic characters, sometimes somewhat ambiguous. In accordance with the results of the present work, other authors have also reported the occurrence of species of the genus *Candida* in association with flowers [37], as well as all other species and/or genera of yeast identified from the sampling of *Q. grandiflora* flowers carried out in this work [13, 23].

In addition to the genus *Candida*, we observed the occurrence of lineages of the genus *Starmerella* in the flowers of *Q. grandiflora*. Corroborating this finding, we verify in the literature that ascomycetic yeast species systematically isolated from ephemeral flowers and their associated insects in neotropical, nearctic, and Australian biogeographic regions belong mainly to the *Starmerella*, *Metschnikowia*, *Kodamaea*, and *Wickerhamiella* clades [15, 38]. Additionally, in agreement with this information, we also identified species of the genera *Kodamaea* and *Wickerhamiella* among the isolates. Many of the genera identified in this study have already been isolated from flowers of other plant species around the world. Some examples are the *Hanseniaspora* and *Rhodotorula* genera, which were also identified by [39] associated with orchid flowers, and the species of the genera *Pichia*, *Meyerozyma*, *Kodamaea*, *Candida*, and *Wickerhamomyces*, also isolated by [40] in flowers of wild and domestic plants. In addition to species of these genera, we isolated the dematiaceous yeast *Exophiala dermatitidis*; it is a species generally found in association with soil and dead plants worldwide [40, 41]; however, we could observe its presence in association with the flowers of *Q. grandiflora*, which increases and corroborates the distribution and occurrence of this species in different habitats. This species is melaninogenicus and commonly associated with phaeohyphomycosis, rarer respiratory and systemic infections in immunocompromised patients, and also with cases of osteomyelitis and septic arthritis [42, 43] and has already been isolated in cherry blossoms.

The two fungal species associated with *Q. grandiflora* and that did not fit phylogenetically as yeasts belong to the genera *Anthracocystis* and *Myrmaecium*. Although these taxa have been identified among yeast isolates and have yeast morphologies, according to [44], the sequences obtained from these yeasts were distributed in different subclades of *Anthracocystis* (Ustilaginales) and none of them can be directly associated with a teleomorphic species. Only one of these yeast anamorphs was assigned to a species, namely, *Pseudozyma flocculosa*. Following the recent practice of fusing anamorphic yeast species with a sexual stage (teleomorphic) under the oldest generic name, this new yeast taxon originated from recombination with *Anthracocystis*, resulting in *Anthracocystis flocculosa*. The phytopathogenic species *Ustilago maydis* and *Ustilago esculenta*, of the same family as *Anthracocystis heteropogonicola* (Ustilaginaceae), are known examples of dimorphic or yeast-like fungi since their cells can develop as yeasts or take on mycelial form according to the environmental signals received [45, 46]. No records of *A. heteropogonicola* growing as a dimorphic fungus were found, which may be a relevant contribution to the description of this species. A similar fact was observed for *Myrmaecium rubrum* (Valsariaceae), as no direct references to its growth as a dimorphic fungus were found. However, it is known that this characteristic exists for other species of the family, as observed by [47] for *Valsaria insitiva*, a phytopathogenic Dothideomycetes that causes perennial cancer in apples and almonds [48].

The isolates JGM1 (*Priceomyces*), PPA10 and PPA21 (*Candida*), PP18 (*Kazachstania*), and PPB13 (*Wickerhamomyces*) could not be identified to the species level. The molecular phylogenetic analyses based on sequences of the D1/D2 region of large subunit (26S) rDNA, as well as the internal transcribed spacer (ITS) region of these strains, showed that three of them fit as possible new species (PPA10, PPA18, and PPA21). According to available data from previously deposited sequences, PPA18 is a possible new species closely related to *Kazachstania servazzii*, *Kazachstania africana*, and *Kazachstania aerobia* (all with 96% similarity). In addition, the isolates PPA10 and PPA21 correspond to a possible new species of the *Clavispora* clade, called *Candida ruelliae* (85% identity) isolated originally from flowers of *Ruellia* sp. [49]. They also have genetic similarity with two other records of the genus *Candida* (both with 97% identity, unpublished data).

Corroborating the findings of the present work and in agreement with reports available in the literature, the genus *Qualea* is a very promising niche for the isolation of new mycological species. This can be seen in the bioprospecting studies carried out by [50], who described two new fungal species of the genus *Uncinula* associated with the leaves of *Q. grandiflora* and *Qualea multiflora*, respectively. In another study, Araújo et al. [51] describe three other new mycological species belonging to the genera *Alternaria*, *Passalora*, and *Periconiella* associated with the leaves of *Q. grandiflora* and *Qualea parviflora*.

Identifying different species of yeasts in *Q. grandiflora* flowers, in addition to reinforcing the status of this plant as a super host for biodiversity [50], is an incentive for conservation at the natural and social scales of the Cerrado biome. As an example, we identified species of the genera *Meyerozyma* and *Wickerhamomyces*. Species of these genera have physiological characteristics that allow them to perform with great performance in the spontaneous fermentation of distilled drinks [52]. This fact is of particular importance, given that the northern region of the state of Minas Gerais has worldwide prominence in the production of artisanal cachaças and sugar cane spirits. In this context, it is extremely desirable to characterize new yeast species in order to select the most efficient and productive strains. In line with the aforementioned data, studies of this nature come as an incentive to further efforts aimed at protecting the Cerrado biome, as well as its associated macro- and microbiodiversity.

**Figure 2:**
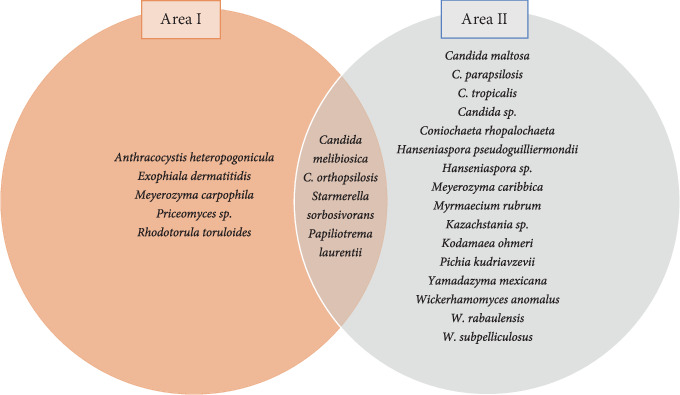
Yeast diversity in flowers of *Q. grandiflora* for each sampled area and shared by both areas (phyla Ascomycota and Basidiomycota).

Based on isolates per sampled area, the number of species was significantly higher for Area II (Figure 2), even considering that the sampling effort was equivalent in both locations. This significant difference in the number of species between the two areas may be correlated with climatic, ecological, socioenvironmental, or land use differences. Despite the more preserved aspect of the surrounding vegetation, Area I had a lower number of yeast species than Area II. Thus, it can be speculated that floral visitors (who act as dispersers of yeasts) of Area II have a limited variety of resources and have adopted generalist habits of foraging. Furthermore, to meet their nutritional and physiological needs, they need to visit a greater variety of plant species, resulting in greater dispersion and favoring an increase in the number of vectorized yeast species. Despite the significant difference in the number of species, some occurred in both areas, suggesting vertical transmission, floral visitors of the same species, or similarities in the floristic composition between the studied regions.

We emphasize that only four of all the species identified were shared between the two areas (Figure 2). Although premature, we deduce that these species may be more evolutionarily associated with *Q. grandiflora* in terms of occurrence in its flowers, response to visiting insects or specialized pollinators, and more related to foraging in that niche. More broadly, the isolates obtained there were separated into ascomycetes and basidiomycetes, with the phylum Ascomycota representing 91.3% of all the identified isolates. Considering that ascomycetic yeasts use the sugars offered by the different floral organs, and that they obtain energy preferably through the fermentative route, this percentage may be related to the supply of nectar and the consequent availability of sugars in the flowers of *Q. grandiflora* [53]. This observation corroborates other studies of yeast diversity in flowers, where the percentage of ascomycete yeasts was substantially higher than that of basidiomycete yeasts [14].

**Figure 3:**
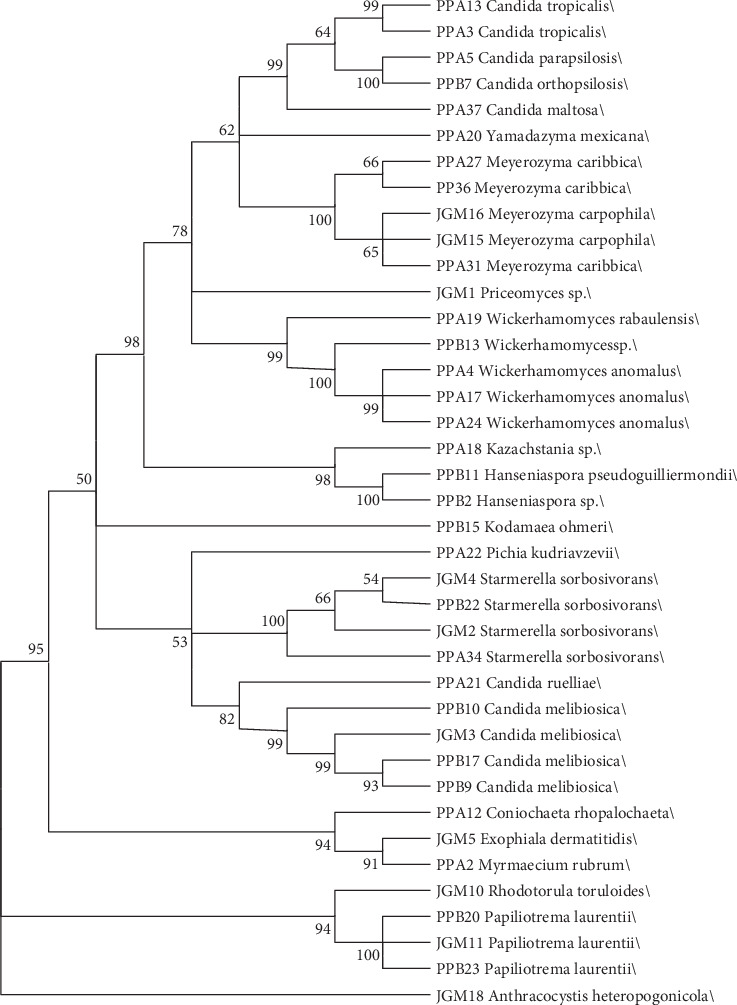
Molecular phylogenetic analysis by maximum likelihood and the Kimura model of two parameters of yeast species related to *Q. grandiflora* flowers, based on the NJ analysis of the sequences of the D1/D2 regions of the large subunit rRNA gene. The bootstrap consensus tree inferred from 1000 replicates is taken to represent the evolutionary history of the taxa analyzed. Branches corresponding to partitions reproduced in less than 50% bootstrap replicates are collapsed. The percentage of replicate trees in which the associated taxa clustered together in the bootstrap test (1000 replicates) are shown next to the branches. ID code numbers for all strings are indicated after the strain name (correspondent GenBank accession numbers is on Table 1). Culture collection prefixes: UFMG-CM (Collection of Microorganisms and Cells from the Federal University of Minas Gerais), BCC (BIOTEC Culture Collection), CBS (yeast collection of the Westerdijk Institute), NRRL Y (ARS Culture Collection), and ATCC (American Type Culture Collection). The tree was drawn to scale, with the lengths of the branches representing the number of substitutions per location. Evolutionary analyses were conducted on MEGA X [54].

In order to demonstrate how the identified species are phylogenetically correlated, a maximum likelihood tree was generated (Figure 3). The highest bootstrap values of the phylogenetic analysis determined clades of isolates significantly corresponding to their respective genera. This demonstrates the phylogenetic proximity among the organisms identified in both sampled areas.

### 3.2. Screening for Extracellular Enzymatic Activity

Table 2 indicates the extracellular enzyme production profiles of the tested yeast isolates. Of these, 44.8% were able to produce at least one of the studied enzymes.

The use of solid media supplemented with specific substrates is a standard methodology for detecting enzyme production by microorganisms. With this method, the radial growth of colonies and hydrolysis halos are measured and, through this direct correlation, the EI is established. Bocchese et al. and Bandeira et al. [55, 56] recommend an EI ≥ 2.0 to show the ability of a microorganism to synthesize enzymes in a solid medium, whereas Ferreira et al. [57] consider strains with EI > 1.50 to be potential producers. Here, like [58], we consider EI ≥ 2.0 to infer the character of potential enzyme producer.

In this context, the best enzyme production among the microorganisms tested was observed with the cellulase test, since among the 20 isolates that presented hydrolysis halos in this test, eight presented EI ≥ 2.0, with values ranging from 2 to 6.44. For amylase production, halo formation was observed in 12 isolates with EIs ranging from 1.33 to 3.6. In the protease assays, EIs ranging from 1.21 to 3.0 were observed among the 12 isolates with some hydrolysis halo formation, while xylanase production was observed in only five isolates with general EIs ranging from 1.5 to 2.12. In the tannase test, enzyme production was also observed in only five isolates; however, the general EIs ranged from 1.5 to 2.0. None of the isolates tested produced pectinolytic enzymes.

When analyzing EIs by substrate tested, the highest values for enzymatic production in the cellulase test were expressed by the fungus *M. rubrum* and the yeast *Papiliotrema laurentii* (6.44 and 5.42, respectively). However, in terms of isolates, the species *Coniochaeta rhopalochaeta* had the highest number with cellulolytic activity. These findings corroborate the data presented by [59] who demonstrated the ability of *Coniochaeta pulveracea* to produce cellulases that degrade cellulose, suggesting that this species and its closest relatives (e.g., *Coniochaeta boothii*, *C. rhopalochaeta*, and *Coniochaeta subcorticalis*) are some of the main yeasts responsible for the hydrolysis of wood given their systems of extracellular hydrolytic enzymes. The EIs expressed by the isolates of *P. laurentii* are interesting since they were all > 2.0. This observation supports the fact that basidiomycetes are the most potent degraders of the cellulose polymer [60].

The highest EIs observed in the amylase test were for an isolate of the species *C. rhopalochaeta* and for an isolate of *M. rubrum*. In the xylanase test, production was low in both the total number of isolates and by individual isolate, with no EI ≥ 2.0. In the tannic acid test, however, it is interesting to note that of the five isolates that showed some EI value, four were of the species *C. rhopalochaeta*. Additionally, among all the isolates that showed some enzymatic activity, *C. rhopalochaeta* proved to be the species with a more heterogeneous application potential, as it was the only species among the tested strains that showed some enzymatic production in all the tests with positive results.

The analysis of enzymatic production by microorganism isolation area revealed that most of the isolates with positive results come from Area II, which can be correlated with the greater number of yeast species from that area (Figure 1). In addition to this variation between isolates by area, there was variation in enzymatic production among strains of the same species. For example, the EI values for the isolates of *C. rhopalochaeta* and *Priceomyces* sp., whose tests showed positive results, varied significantly. For this reason, regarding the data used from the *Coniochaeta* isolates, each one was isolated from different floral samples; for this reason, their enzyme profile was studied individually.

Some studies have demonstrated the ability of microbial strains to produce enzymes with potential for industrial use, and hydrolytic enzymes such as cellulases and amylases have stood out [61]. Although cellulases are produced especially by bacteria and filamentous fungi [62], we can see that epiphytic yeast isolated from flowers of *Q. grandiflora* also has the ability to synthesize enzymes of this complex. In addition, some studies with yeasts isolated from different substrates, such as flowers [63], fruits [61, 64], and decomposing leaves [65], have shown the potential that this group of microorganisms has for the production of cellulase.

Amylases and proteases form one of the main groups of industrial enzymes, given their broad spectrum of biotechnological applications [66]. Amylases are responsible for the hydrolysis of starch into maltose, glycogen, dextrins, and progressively smaller polymers composed of glucose units [67] and are closely related to fruit ripening and sweet taste. Proteases, on the other hand, are responsible for the proteolytic cleavage of peptides. Proteolytic enzymes produced by microorganisms can act in the hydrolysis of proteins of the cell membrane and wall of the host plants, facilitating penetration and infection [68]. In the present study, we observed that, among the strains with some enzymatic expression, the isolates of the species *C. rhopalochaeta* were the best producers of both amylase and protease, with emphasis on the amylolytic synthesis by the PPB4 isolate, which had an EI well above the reference value for potential producers.

Xylanases produced by microorganisms, as well as proteases, are commonly related to phytopathogens, since they have the ability to decompose hemicellulose, which is one of the main components of the plant cell wall [69]. Tannin acyl hydrolase or tannase, in turn, catalyzes the hydrolysis of tannins by producing gallic acid and glucose [70]. Tannins are part of the plant defense system against microorganisms, so the production of tannase can be considered a microbial counterattack [71]. Our results revealed that only 8.62% of the tested yeast isolates expressed some enzymatic production in the xylanase and tannase tests, respectively. This low enzymatic productivity may be related to the isolation loci of the microorganisms [72], as they were isolated from the surface of flowers and not from internal parts, as expected for enzyme-secreting microorganisms involved in the process of phytopathogenesis.

In addition to demonstrating one of the biochemical interfaces of the studied group of microorganisms, the analysis of the data obtained for the production of the aforementioned enzymes subsidizes knowledge of microbial bioprospecting for enzyme synthesis, which is an area under increasing expansion. Furthermore, despite the fact that enzymes from traditional sources, such as animals and plants, have greater acceptance in the world market, recent years have seen a trend to replace these enzyme sources with microbial sources, given not only their susceptibility to genetic manipulation but also their broad biochemical diversity [19]. Enzymes obtained by natural means, in addition to being less financially costly, are more easily approved by regulatory agencies than those obtained by synthetic or genetically modified routes [73]. Despite the fact that filamentous fungi are more exploited in terms of enzyme synthesis, yeasts have advantages over them because they are easy to handle, fast-growing, do not have great nutritional needs, and the cost of materials for cultivating them is less [74]. In addition, they are likely to present greater production through genetic manipulation, and their enzymes have the ability to catalyze various reactions, giving them not only biochemical versatility but also more specific application conditions [49, 75].

From this perspective, the search for new yeast strains in tropical biomes that produce enzymes is essential for the area of bioprocesses. In addition, the discovery of wild yeast lineages with potential for enzymatic production means finding new microbial functions for the described taxa, so that it is fully possible and desirable to expand the use of microorganisms in new bioassays aiming at directional selection, that is, as a form of wild species screening [76, 77]. While culture-independent methods have the potential to offer a clearer and more accurate understanding of yeast diversity in tropical forest biomes—and to determine if these ecosystems host greater diversity than temperate forests—research in this area remains limited. Despite the rich variety of habitats and intricate ecological interactions characteristic of tropical forests, relatively few studies have explored their yeast biodiversity [78]. Yeasts and yeast-derived products exhibit broad applicability across a wide range of industrial and biotechnological sectors. These include the production of fermented foods and beverages, pharmaceuticals, industrial enzymes, vitamins, carotenoids, lipids, steroids, polysaccharides, *β*-glucans, nucleotides, flavor compounds, biofuels, and various platform chemicals—also referred to as chemical building blocks. Moreover, yeasts play a role in environmental applications such as biocontrol and bioremediation [79].

## 4. Conclusions

This study investigating the yeast diversity in flowers of *Q. grandiflora*, a native plant of Brazil's Cerrado biome, analyzed 96 floral samples and isolated 58 yeast strains, comprising 56 true yeasts and two yeast-like fungi. These isolates were classified into 18 distinct yeast species across 14 genera, with *Candida* being the most prevalent, represented by five species. Other notable genera included *Meyerozyma*, *Wickerhamomyces*, *Starmerella*, *Coniochaeta*, *Kodamaea*, and *Wickerhamiella*. Additionally, the dematiaceous yeast *E. dermatitidis*, typically associated with soil and decaying plant matter, was identified. Two isolates, PPA10 and PPA21, showed 85% identity to *C. ruelliae*, suggesting the presence of a potentially new species within the *Clavispora* clade.

The study also assessed the enzymatic potential of these isolates, focusing on hydrolytic enzymes such as cellulase, amylase, and protease. In cellulase activity assays, 20 isolates formed hydrolysis halos, with eight exhibiting an EI of ≥ 2.0, ranging from 2 to 6.44. The highest cellulase activity was observed in the fungus *M. rubrum* and the yeast *P. laurentii*, with EIs of 6.44 and 5.42, respectively. *C. rhopalochaeta* had the highest number of isolates displaying cellulolytic activity. In amylase assays, the highest EIs were recorded for isolates of *C. rhopalochaeta* and *M. rubrum*. However, xylanase production was low across all isolates, with no EI ≥ 2.0 observed. Notably, *C. rhopalochaeta* isolates were the most proficient producers of both amylase and protease, particularly the PPB4 isolate, which showed significant amylolytic activity.

These findings suggest that, through genetic manipulation, these yeasts could enhance enzyme production, offering biochemical versatility and potential for specific industrial applications. This research underscores the ecological significance of flowers as niches for diverse yeast communities and highlights the biotechnological potential of yeasts associated with the Cerrado biome. The discovery of potentially new species and their enzymatic capabilities emphasizes the importance of conserving this biome and exploring its microbial diversity for sustainable biotechnological applications.

Finally, mapping new habitats of biodiversity is of great importance not only for knowledge and applicability but mainly for the conservation of these *hotspots*.

## Figures and Tables

**Table 1:** Diversity of yeast microorganisms isolated from *Qualea grandiflora*. The species are represented in the table, together with their identification codes (ID), lineage codes, accession numbers of each strain, D1/D2 accession number, and the percentages of identity of the strings in GenBank. The accession numbers to the strains(⁣^∗^) are those of the Collection of Microorganisms and Cells of the Federal University of Minas Gerais (UFMG-CM), BIOTEC Culture Collection (BCC), yeast collection from the Westerdijk Institute (CBS), Culture Collection ARS (NRRL Y), and American Type Culture Collection (ATCC).

**Code ID**	**Species or genus**	**Strain number**⁣^∗^	**Accession number**	**D1/D2 accession number**	**% ID**	**Phylum**
JGM18	*Anthracocystis heteropogonicola*	CBS 312.66	MH870442.1	MT543180.1	100%	Basidiomycota
PPA37	*Candida maltosa*	CBS:5611	KY106554.1	MT543195.1	99%	Ascomycota
PPB17	*Candida melibiosica*	CR81	MK110080.1	MT543203.1	100%	Ascomycota
JGM3	*Candida melibiosica*	NRRL Y-17076	NG_055230.1	MT543177.1	100%	Ascomycota
PPB9	*Candida melibiosica*	NRRL Y-17076	NG_055230.1	MT543209.1	100%	Ascomycota
PPB10	*Candida melibiosica*	NRRL Y-17076	NG_055230.1	MT543199.1	100%	Ascomycota
PPB6	*Candida orthopsilosis*	ATCC 96139	NG_054816.1	MT543208.1	99%	Ascomycota
PPB7	*Candida orthopsilosis*	ATCC 96139	NG_054816.1	MT543208.1	99%	Ascomycota
JGM9	*Candida orthopsilosis*	CBS:10906	KY106633.1	MT543208.1	98%	Ascomycota
PPA5	*Candida parapsilosis*	ATCC 22019	NG_054833.1	MT543198.1	99%	Ascomycota
PPA10	*Candida* sp./*Candida* sp./*[Candida] ruelliae*	NRRL Y-27702/UFMG-CM-Y6408/MTCC 7739	AY640205.1/MH379817.1/NG_064309.1	MT543187.1	97%/97%/85%	Ascomycota
PPA21	*Candida* sp./*Candida* sp./*[Candida] ruelliae*	NRRL Y-27702/UFMG-CM-Y6408/MTCC 7739	AY640205.1/MH379817.1/NG_064309.1	MT543187.1	97%/97%/85%	Ascomycota
PPA3	*Candida tropicalis*	ATCC 750	NG_054834.1	MT543196.1	99%	Ascomycota
PPA13	*Candida tropicalis*	ATCC 750	NG_054834.1	MT543182.1	100%	Ascomycota
PPA1	*Coniochaeta rhopalochaeta*	CBS 109872	GQ351561.1	MT543181.1	99%	Ascomycota
PPA7	*Coniochaeta rhopalochaeta*	CBS 109872	GQ351561.1	MT543181.1	99%	Ascomycota
PPA8	*Coniochaeta rhopalochaeta*	CBS 109872	GQ351561.1	MT543181.1	99%	Ascomycota
PPA9	*Coniochaeta rhopalochaeta*	CBS 109872	GQ351561.1	MT543181.1	99%	Ascomycota
PPA11	*Coniochaeta rhopalochaeta*	CBS 109872	GQ351561.1	MT543181.1	99%	Ascomycota
PPA12	*Coniochaeta rhopalochaeta*	CBS 109872	GQ351561.1	MT543181.1	99%	Ascomycota
PPA33	*Coniochaeta rhopalochaeta*	CBS 109872	GQ351561.1	MT543181.1	99%	Ascomycota
PPB4	*Coniochaeta rhopalochaeta*	CBS 109872	GQ351561.1	MT543181.1	99%	Ascomycota
JGM5	*Exophiala dermatitidis*	CBS 129514	MH878058.1	MT543179.1	99%	Ascomycota
PPB2	*Hanseniaspora guilliermondii/H. opuntiae*	CBS:465/CBS 8733	KY107797.1/NG_055312.1	MT543207.1	99%/99%	Ascomycota
PPB11	*Hanseniaspora pseudoguilliermondii*	CBS 8772	NR_155181.1	MT543200.1	99%	Ascomycota
PPA27	*Meyerozyma caribbica*	CBS 9966	MK394109.1	MT543190.1	100%	Ascomycota
PPA31	*Meyerozyma caribbica*	CBS 9966	MK394109.1	MT543192.1	100%	Ascomycota
PPA36	*Meyerozyma caribbica*	CBS 9966	MK394109.1	MT543194.1	99%	Ascomycota
JGM15	*Meyerozyma carpophila*	CBS 5256	MK394110.1	MT543173.1	100%	Ascomycota
JGM16	*Meyerozyma carpophila*	CBS 5256	MK394110.1	MT543174.1	99%	Ascomycota
PPA2	*Myrmaecium rubrum*	CBS 345.86	MH873654.1	MT543191.1	98%	Ascomycota
PPA18	*Kazachstania servazzii/K. africana/K. aerobia*	NRRL Y-12661/CBS 2517/CBS 9918	NG_055029.1/XR_002430158.1/NG_055042.1	MT543184.1	96%/96%/96%	Ascomycota
PPB15	*Kodamaea ohmeri*	CBS 5367	MK394144.1	MT543202.1	100%	Ascomycota
PPB20	*Papiliotrema laurentii*	CBS 139	NG_056281.1	MT543204.1	99%	Basidiomycota
JGM11	*Papiliotrema laurentii*	CBS 139	NG_056281.1	MT543172.1	99%	Basidiomycota
PPB23	*Papiliotrema laurentii*	CBS 139	NG_056281.1	MT543206.1	99%	Basidiomycota
PPA22	*Pichia kudriavzevii*	CBS5147	CP028532.1	MT543188.1	100%	Ascomycota
JGM1	*Priceomyces* sp./*P. melissophilus*	UFMG-CM-Y6328/CBS:6344	MG737686.1	MT543175.1	100%/99%	Ascomycota
JGM10	*Rhodotorula toruloides*	CBS:12050	KY109171.1	MT543171.1	100%	Basidiomycota
JGM2	*Starmerella sorbosivorans*	CBS 8768	NG_060827.1	MT543176.1	99%	Ascomycota
JGM4	*Starmerella sorbosivorans*	CBS 8768	NG_060827.1	MT543178.1	99%	Ascomycota
JGM14	*Starmerella sorbosivorans*	CBS 8768	NG_060827.1	MT543178.1	99%	Ascomycota
PPA15	*Starmerella sorbosivorans*	CBS 8768	NG_060827.1	MT543178.1	99%	Ascomycota
PPA32	*Starmerella sorbosivorans*	CBS 8768	NG_060827.1	MT543178.1	99%	Ascomycota
PPA34	*Starmerella sorbosivorans*	CBS 8768	NG_060827.1	MT543193.1	99%	Ascomycota
PPA35	*Starmerella sorbosivorans*	CBS 8768	NG_060827.1	MT543193.1	99%	Ascomycota
PPA38	*Starmerella sorbosivorans*	CBS 8768	NG_060827.1	MT543178.1	99%	Ascomycota
PPB21	*Starmerella sorbosivorans*	CBS 8768	NG_060827.1	MT543178.1	99%	Ascomycota
PPB22	*Starmerella sorbosivorans*	CBS 8768	NG_060827.1	MT543205.1	100%	Ascomycota
PPA4	*Wickerhamomyces anomalus*	CBS:5759	KY110078.1	MT543197.1	100%	Ascomycota
PPA17	*Wickerhamomyces anomalus*	CBS:5759	KY110078.1	MT543183.1	100%	Ascomycota
PPA24	*Wickerhamomyces anomalus*	CBS:5759	KY110078.1	MT543189.1	100%	Ascomycota
PPA30	*Wickerhamomyces anomalus*	CBS:5759	MK394130.1	MT543197.1	99%	Ascomycota
PPA39	*Wickerhamomyces anomalus*	CBS:5759	MK394130.1	MT543197.1	99%	Ascomycota
PPA40	*Wickerhamomyces anomalus*	CBS:5759	MK394130.1	MT543197.1	99%	Ascomycota
PPA19	*Wickerhamomyces rabaulensis*	NRRL Y-7945	NG_057165.1	MT543185.1	100%	Ascomycota
PPB13	*Wickerhamomyces subpelliculosus/W. anomalus*	NRRL Y-1683/CBS:5759	NG_057173.1/KY110078.1	MT543201.1	99%/99%	Ascomycota
PPA20	*Yamadazyma mexicana*	CBS 7066	NG_058439.1	MT543186.1	100%	Ascomycota

**Table 2:** Extracellular enzymatic activity of yeast microorganisms isolated from *Q. grandiflora* flowers, estimated using the enzymatic index (EI). The table shows only the isolates that showed an enzymatic profile for at least one substrate source.

**Code**	**Species**	**Cellulase**	**Amylase**	**Xylanase**	**Protease**	**Tannase**	**Pectinase**
JGM5	*Priceomyces* sp.	—	—	—	1.3	—	—
JGM10	*Priceomyces* sp.	—	—	—	1.21	2	—
JGM11	*Papiliotrema laurentii*	2.75	—	—	—	—	—
JGM18	*Anthracocystis heteropogonicola*	2	1.81	—	—	—	—
PPA1	*Coniochaeta rhopalochaeta*	1.85	1.42	—	1.21	—	—
PPA2	*Myrmaecium rubrum*	6.44	3.6	1.66	—	—	—
PPA3	*Candida tropicalis*	—	—	2.12	—	—	—
PPA7	*Coniochaeta rhopalochaeta*	2.10	2.14	—	1.23	—	—
PPA8	*Coniochaeta rhopalochaeta*	3.25	2.33	—	1.72	1.5	—
PPA9	*Coniochaeta rhopalochaeta*	1.89	—	—	1.22	—	—
PPA11	*Coniochaeta rhopalochaeta*	2.88	2.27	—	1.38	1.5	—
PPA12	*Coniochaeta rhopalochaeta*	1.68	1.85	—	1.25	—	—
PPA13	*Candida tropicalis*	—	—	2.12	—	—	—
PPA20	*Yamadazyma mexicana*	1.2	—	—	—	—	—
PPA24	*Wickerhamomyces anomalus*	1.87	1.33	—	—	—	—
PPA30	*Wickerhamomyces anomalus*	1.75	2.33	—	—	—	—
PPA33	*Coniochaeta rhopalochaeta*	1.8	1.8	1.5	1.45	2	—
PPA35	*Starmerella sorbosivorans*	1.61	—	—	1.44	—	—
PPA38	*Starmerella sorbosivorans*	—	—	1.5	—	—	—
PPA40	*Wickerhamomyces anomalus*	1.25	2	—	—	—	—
PPB4	*Coniochaeta rhopalochaeta*	2.08	6.66	—	1.51	1.85	—
PPB11	*Hanseniaspora pseudoguilliermondii*	1.25	—	—	—	—	—
PPB13	*Wickerhamomyces subpelliculosus*	1.25	—	—	—	—	—
PPB17	*Candida melibiosica*	—	—	—	1.28	—	—
PPB20	*Papiliotrema laurentii*	5.62	—	—	—	—	—
PPB23	*Papiliotrema laurentii*	5	—	—	—	—	—

## Data Availability

The data that support the findings of this study are available from the corresponding author upon reasonable request.
